# Advancements in Surgical Management of Periacetabular Metastases: Emphasizing Minimally Invasive Techniques

**DOI:** 10.3390/cancers17061015

**Published:** 2025-03-18

**Authors:** Jian Guan, Feiyang Qi, Haijie Liang, Xingyu Liu, Zhiqing Zhao, Linxi Chen, Ranxin Zhang, Ryan Y. Yang, Barlas Goker, Swapnil Singh, Bang H. Hoang, David S. Geller, Jichuan Wang, Rui Yang

**Affiliations:** 1 Musculoskleletal Tumor Center, Beijing Key Laboratory for Musculoskeletal Tumors, Peking University People’s Hospital, Beijing 100044, China; gjian04@i.smu.edu.cn (J.G.); 1910301241@pku.edu.cn (F.Q.); lianghaijie@pku.edu.cn (H.L.); lxysunknight@bjmu.edu.cn (X.L.); zhaozhiqing@pku.org.cn (Z.Z.); 2411110402@bjmu.edu.cn (L.C.); 2 The First School of Clinical Medicine, Nanfang Hospital Southern Medical University, Guangzhou 518060, China; 3 Department of Orthopedic Surgery, Montefiore Medical Center, Albert Einstein College of Medicine, Bronx, NY 10461, USA; ranxin.zhang@einsteinmed.edu (R.Z.); ryan@einsteinmed.edu (R.Y.Y.); barlas.goker@nyulangone.org (B.G.); ssingh1@sbhny.org (S.S.); bahoang@montefiore.org (B.H.H.); dgeller@montefiore.org (D.S.G.)

**Keywords:** periacetabular metastasis, minimally invasive reconstruction, osteolytic lesion, tripod technique, quality of life

## Abstract

This review aims to summarize the evolution of surgical techniques for periacetabular metastatic cancer, assess their strengths and limitations, and clarify the corresponding indications. We conducted a comprehensive literature review on periacetabular metastatic cancer, summarizing surgical techniques involving both open and minimally invasive approaches. Additionally, we evaluated the indications for different minimally invasive techniques and proposed potential combinations of these techniques. Our review underscores the benefits of minimally invasive surgery, including reduced surgical trauma, improved patient mobility, lower complication rates, and expedited recovery times, facilitating earlier initiation of systemic cancer therapies. These techniques show substantial potential for broader application in the future. Despite the historical reliance on open surgery as the standard treatment, minimally invasive approaches are emerging as a promising alternative, particularly for managing osteolytic metastases around the acetabulum. This review provides insights into the optimal integration of these techniques, aiming to support evidence-based clinical decision-making and improve patient outcomes.

## Simple Summary

The surgical management of periacetabular metastases has significantly evolved with advancements in both open and minimally invasive techniques. Selecting the most appropriate surgical approach is crucial for improving patient outcomes. This review summarizes the development and benefits of these techniques, with a particular focus on minimally invasive approaches, which offer advantages such as reduced surgical trauma, faster recovery, and lower complication rates. By providing a comprehensive overview of their applications combinations and specific indications, this review aims to guide clinicians in optimizing treatment strategies for patients with periacetabular metastatic cancer.

## 1. Introduction

Advances in cancer treatment have led to improved survival rates, resulting in a concurrent increase in patients presenting with metastatic disease, with the skeleton being among the most frequently affected organs [1,2]. The spine, pelvic bones, proximal femur, and humerus are particularly susceptible to metastases, with the pelvis, specifically the periacetabular region, being the second most common site for metastasis [3,4]. Most patients with bone metastases develop skeletal-related events (SREs), including ostealgia, pathological fractures, hypercalcemia, and bone marrow suppression. These complications often lead to decreased mobility, prolonged bed confinement, and disruptions in systemic cancer treatment. Consequently, the management of periacetabular metastases presents a clinical challenge.

Traditionally, non-surgical treatments, including pain management and radiation therapy, have been widely used for symptomatic acetabular lesions, primarily aimed at symptom relief [5]. However, due to the persistent invasion of the tumor into bone tissue and subsequent compromised mechanical properties, pain control often remains palliative, and long-term use of analgesics is associated with significant side effects [6,7]. Radiation therapy can provide localized tumor control and pain relief, but the periacetabular bone is deeply embedded within the soft tissue capsule, surrounded by vital lymphatic vessels, neurovascular structures, and multiple muscle attachment sites. Despite recent advances in high-precision radiotherapy techniques, complications such as lower limb lymphedema and hip joint stiffness remain common [8,9,10]. Furthermore, radiotherapy alone is often inadequate for managing existing pathological fractures, further highlighting the necessity for surgical intervention.

Open surgical procedures, including tumor curettage, bone cement augmentation, and total hip reconstruction with acetabular prostheses, have long been the primary surgical strategies. While these techniques effectively relieve pain and restore function, they are associated with high complication rates, including significant intraoperative blood loss, periprosthetic infection or failure, and delayed wound healing. Approximately up to 49.6% of patients may experience complications, with one-fifth having severe complications [2,3,4]. Additionally, patients with advanced metastatic cancer are typically older and often have comorbidities, and may not be ideal candidates for open surgery due to its inherent surgical trauma. As a result, only those with favorable survival expectations or oligo bone metastasis are possible candidates for open surgical interventions [2,11].

In recent years, minimally invasive surgical techniques have gained popularity as alternative treatment strategies for periacetabular metastases [12]. These approaches offer several advantages, including reduced surgical burden, faster recovery, and fewer complications, enabling earlier resumption of systemic therapies. By providing stable reconstruction to maintain functionality, minimally invasive techniques have demonstrated significant potential for a broader range of patients, particularly those with limited physical fitness [12,13]. Current minimally invasive interventions often involve tumor ablation followed by structural reinforcement and have shown favorable clinical outcomes. As these techniques continue to evolve, they represent a transformative shift in the surgical management of periacetabular metastases [12].

## 2. Necessity for Surgical Intervention: Clinical Manifestation of Periacetabular Metastatic Lesions

### 2.1. Ostealgia

The acetabulum, a pivotal anatomical structure, plays an essential role in force transmission between the upper body and lower limbs. The development of metastatic cancer in the periacetabular region often marks a critical progression in disease severity [2,11]. Ostealgia is the most common clinical symptom of bone metastasis, frequently presenting as pain radiating to the back and lower limbs. Initially, ostealgia may present as intermittent but often progresses to persistent pain, particularly when mechanical abnormalities are present in the bone [4]. Analgesics and narcotics are commonly used in treating patients with periacetabular metastases, often requiring high doses. However, prolonged opioid use can lead to dependency in over 60% of patients and is associated with considerable side effects, making long-term management challenging [14,15,16]. Additionally, treatments such as bisphosphonates and denosumab have been effective in alleviating ostealgia in over 50% of patients, offering partial relief from metastatic bone pain [5,6,7].

### 2.2. Pathological Fractures and Acetabular Instability

Pathological fractures are among the initial events experienced by many patients with bone metastasis, often signifying advanced bone destruction. Although microfractures may occur before clinical symptoms become apparent, they are generally undetectable at early stages [11,17]. The primary causes of pathological fractures include osteolysis induced by the tumor, paraneoplastic syndrome, and cachexia associated with the primary malignancy. These fractures typically present with severe pain and impaired hip joint mobility, often resulting in prolonged immobility and increased long-term mortality [18]. In symptomatic periacetabular patients, the emphasis should be on preventing severe pathological fractures and managing the underlying disease [19]. When fractures do occur, surgical intervention is generally accepted as an effective means of restoring function and alleviating symptoms.

### 2.3. Hypercalcemia

Hypercalcemia is a common metabolic complication of malignant bone metastases, affecting approximately 30% of patients [2,11,12,13]. It is characterized by elevated serum calcium levels due to abnormal bone formation and resorption processes driven by malignancy. Hypercalcemia in metastatic disease is mediated through various factors, such as parathyroid hormone-related protein (PTHrP), interleukin-1 (IL-1), IL-6, tumor necrosis factor-alpha (TNF-α), and colony-stimulating factors (CSFs). Systemic secretion of PTHrP by malignant tumors, known as humoral hypercalcemia of malignancy (HHM), accounts for 80% of cases [20]. Clinical symptoms primarily affect the kidneys, gastrointestinal tract, and nervous system [16]. In managing hypercalcemia, bisphosphonates or denosumab are primarily used, with calcitonin being an additional option in certain cases [21]. Addressing hypercalcemia is essential for stabilizing patients and preventing systemic complications.

## 3. Rationale for Surgical Intervention: Biology of Osteolytic Metastases

Most metastatic cancer-induced lesions in the acetabulum are osteolytic or mixed, commonly arising from primary cancers of the breast, lung, or kidney [3,4,22]. Osteolytic metastases are primarily characterized by the invasion of tumor cells into bone tissue, leading to alterations in the bone microenvironment through interactions with osteoclasts and osteoblasts, resulting in the disruption or replacement of stable trabecular bone structures. This ultimately leads to biomechanical failure and the onset of clinical symptoms.

In healthy bone, remodeling depends on the balanced activity of osteoblasts (responsible for bone formation) and osteoclasts (responsible for bone resorption). However, in osteolytic metastases, interactions between tumor cells and bone disrupt this balance [23]. Tumor cells overproduce osteolytic factors such as PTHrP, IL-11, IL-6, IL-8, vascular endothelial growth factor (VEGF), tumor necrosis factor (TNF), Jagged 1, and epidermal growth factor (EGF)-like ligands [24,25]. These factors stimulate osteoblasts to express receptor activators of nuclear factor-κB ligand (RANKL) while downregulating osteoprotegerin (OPG) [22,26]. This imbalance enhances osteoclast activity, leading to excessive bone resorption (osteolysis). This process releases calcium ions, transforming growth factor-β (TGF-β), and insulin-like growth factor 1 (IGF1), all of which promote tumor homing, angiogenesis, tumor proliferation, and further release of PTHrP, thereby perpetuating a “vicious cycle” [22,27].

**Figure 1:**
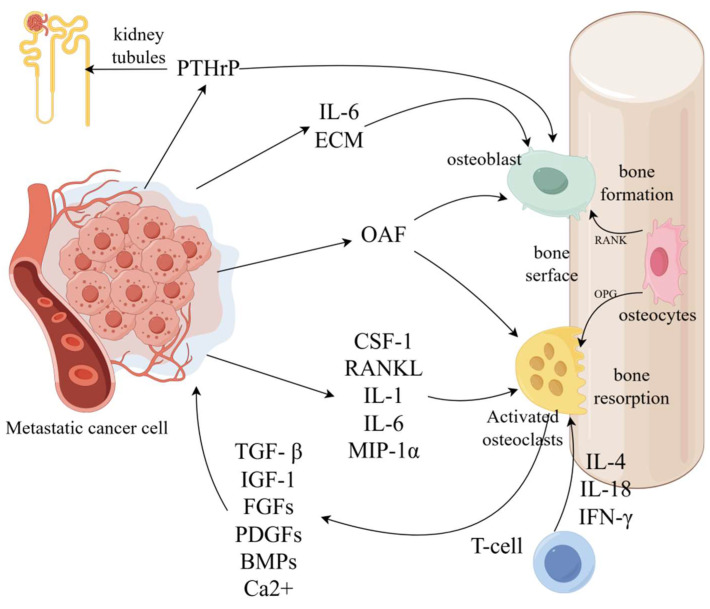
Mechanisms underlying osteolytic metastatic lesions. PTHrP, parathyroid hormone-related peptide; ECM, extracellular matrix; RANKL, receptor activator of NF-κB ligand; RANK, receptor activator of NF-κB; OPG, osteoprotegerin; OAF, osteoclast activating factors; CSF-1, colony-stimulating factor-1; MIP-1α, macrophage inflammatory protein-1α; IFN-γ, interferon-γ; TGF-β, transforming growth factor-β; IGF-1, insulin-like growth factor-1; FGFs, fibroblast growth factors; PDGFs, platelet-derived growth factor; BMPs, bone morphogenetic proteins; IL, interleukin (Produced by Figdraw).

Bone colonization by metastatic cancer also involves creating a supportive microenvironment that interacts with various cell types, including stromal cells, mesenchymal stem cells, and immune cells [22]. Under the influence of metastatic cancer cells, bone matrix factors such as osteopontin (OPN), osteonectin (ON), periostin (POSTN), bone sialoprotein (BSP), dentin matrix acidic phosphoprotein 1 (DMP1), and syndecan 1 are released to promote bone marrow cell recruitment, tumor migration, and survival [22,26,28]. Furthermore, adipose-derived stem cells (ASCs), under the influence of metastatic cancer cells, produce factors like C-C motif chemokine ligand 5 (CCL5) and alpha-smooth muscle actin (α-SMA), which facilitate tumor angiogenesis and promote cancer progression [29]. Additionally, immune cells within the microenvironment—particularly pro-inflammatory CD4 T cells—secrete RANKL, TNF, and TGF-β, which activate osteoclasts, enhance bone resorption, and drive tumor progression in bone, further contributing to the vicious cycle [26]. Therefore, surgical interventions that aim to eliminate tumor cells resistant to systemic therapies, such as radiotherapy and chemotherapy, could benefit patients with osteolytic metastases. However, further clinical observations and studies are necessary to validate these approaches (Figure 1).

## 4. Planning for Surgical Intervention: Goals and Clinical Classification of Periacetabular Lesions

The objectives of surgical intervention for periacetabular metastatic lesions are to reduce tumor burden, alleviate pain, reinforce the structure of osteolytic metastases, and restore mobility, thereby facilitating the continuation or initiation of systemic cancer therapies. Surgical strategies should prioritize faster recovery, minimize complications, and streamline treatment processes, including outpatient procedures where feasible.

**Figure 2:**
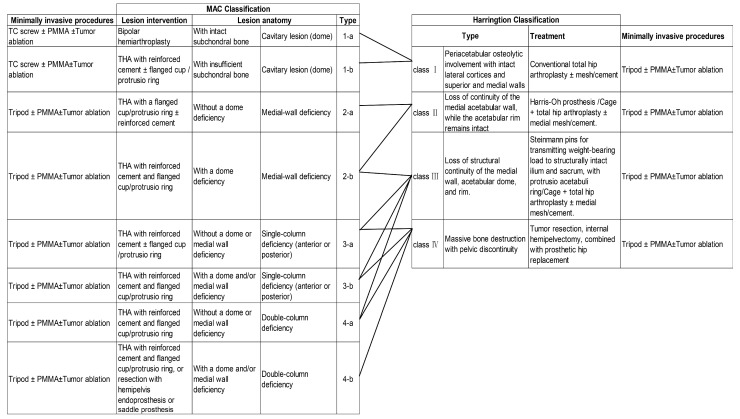
Comparison of MAC and Harrington classification systems and associated interventions.

Effective surgical planning requires a comprehensive classification system to evaluate the anatomical and mechanical impact of metastatic lesions. These classification systems guide the selection of appropriate surgical approaches based on the lesion location, the extent of osteolysis, and its effect on acetabular force transmission. In 1981, Harrington proposed a classification based on the integrity of hip bone structures necessary for total hip replacement and the extent of osteolysis caused by the tumor. This system divides patients into four categories: **Class I** (intact lateral cortex and upper and medial walls), **Class II** (medial wall insufficiency), **Class III** (absence of both lateral cortex and superior wall), and **Class IV** (lesion extending into the hemipelvis) [30]. More recently, the Metastatic Acetabular Classification (MAC) system was introduced in 2018. This system evaluates the extent of osteolysis and its impact on the acetabular mechanical structure by dividing the acetabulum into four anatomical regions: the dome, medial wall, anterior column, and posterior column [31,32]. Each region is associated with specific reconstruction strategies, making the MAC system a comprehensive and flexible framework for managing periacetabular lesions. The MAC system incorporates a broader range of surgical approaches, including both traditional open and minimally invasive techniques, thus providing a more holistic approach to surgical planning and patient stratification (Table 1) (Figure 2).

## 5. Execution of Surgical Intervention: Tumor Elimination and Structural Reinforcement

Local oncological surgical interventions for periacetabular metastatic lesions can be broadly categorized into open surgery, involving tumor resection and prosthetic reconstruction, and minimally invasive surgery, focusing on tumor ablation followed by acetabular reinforcement. Each approach has unique advantages, limitations, and indications, emphasizing the importance of tailoring treatment plans based on individual patient needs, tumor biology, and pathological characteristics.

### 5.1. Open Surgery

Open surgical interventions typically involve extensive or intratumoral tumor resection, often accompanied by reconstruction to restore the mechanical stability of the acetabulum. Common procedures include tumor curettage and prosthetic reconstructions, such as total hip arthroplasty (THA) with or without acetabular prostheses, modular hemipelvic prostheses, or custom-made implants. Structural reinforcement techniques, such as bone cement augmentation, screws, and K-wires, are often incorporated to enhance the biomechanical stability of the affected region [33,34,35,36,37]. Although open surgery offers significant pain relief and improved functionality, it is associated with considerable risks. The average operation lasts 4.5 h (ranging from 2.5 to 9.5 h) and involves substantial intraoperative blood loss (average: 1800 mL; range: 300–6000 mL) [38,39,40,41]. Complication rates vary widely (8–60%), with common issues including wound infections (10–20%) and graft-related complications, such as prosthetic dislocations and fixation failures (~10%) [2,38,39,40,41,42,43,44].

Postoperative outcomes are generally favorable but gradual. The Visual Analog Scale (VAS) for pain typically improves, with 70–90% of patients reporting pain relief or reduction [39,45,46]. Recovery is often prolonged; patients may require 4–6 weeks of bed rest to regain mobility. Six months post-surgery, the average Musculoskeletal Tumor Society (MSTS) score is around 65%, with 50–80% of patients showing improved mobility, accompanied by a corresponding improvement in the Eastern Cooperative Oncology Group (ECOG) performance status [2,42,43,44,45,46]. Local tumor progression rates following open surgery range from 0.7% to 46%, depending on the extent of tumor removal [43,47,48]. Notably, wide resections tend to have lower recurrence rates compared to marginal or intralesional resections [49]. Retrospective data suggest that, for appropriately selected patients, the reoperation-free survival rate is approximately 70% (ranging from 40% to 99%) one year after surgery [40,46,49].

Given these challenges, patient selection must be highly strategic and individualized. Open surgery is generally reserved for patients with oligometastatic disease involving the pelvis who are capable of tolerating major surgery and have a relatively long expected survival. In rare cases, where extensive osteolytic lesions compromise the load-bearing function of the acetabulum, prosthetic reconstruction may be the only viable option for providing mechanical support. Furthermore, in patients with large, painful soft tissue masses or resistance to systemic therapies, open surgery may be considered a necessary intervention for palliative symptom relief.

### 5.2. Minimally Invasive Procedures

Minimally invasive surgical techniques have gained prominence as alternatives to open surgery, particularly for patients with comorbidities or limited physical fitness. These procedures encompass tumor ablation to reduce the disease burden, structural reinforcement to restore mechanical stability, or a combination of both approaches. They offer advantages such as reduced surgical trauma, faster recovery, and lower complication rates, thereby expanding treatment options to a broader range of patients [12,13].

#### 5.2.1. Tumor Elimination Techniques

**Figure 3:**
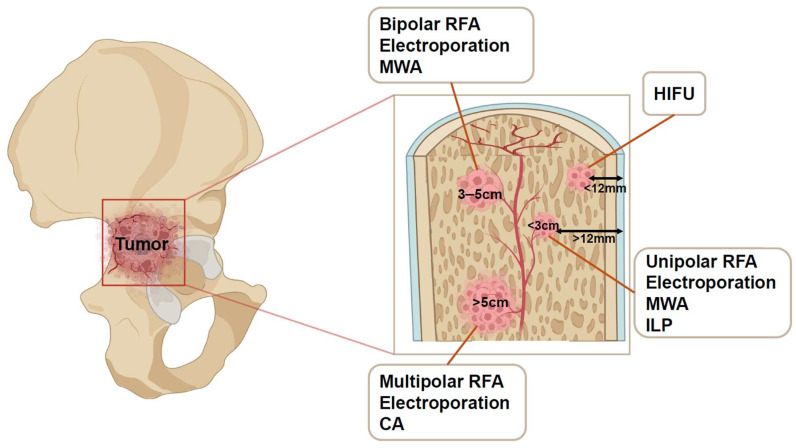
Selection of minimal ablation techniques based on tumor characteristics. The ablation technique chosen for periacetabular metastases depends on the size, shape, and proximity to critical structures. RFA, radiofrequency ablation; MWA, microwave ablation; CA, cryoablation; ILP, interstitial laser photocoagulation; HIFU, high-intensity focused ultrasound.

A variety of tumor ablation techniques are employed, tailored to lesion size, location, and proximity to critical structures (Figure 3).

##### Radiofrequency Ablation (RFA)

RFA employs high-frequency alternating current to induce thermal coagulative necrosis within tumor tissues. The process involves electromagnetic field fluctuations that generate heat through rapid ion vibration, increasing tissue temperatures to 50–100 °C. As temperatures exceed 100 °C, tissue desiccation halts further current flow, limiting the ablation zone to approximately 3 cm. Although larger lesions can be treated with multiple electrodes, the practical size limit is around 5 cm. RFA has been shown to significantly reduce pain in more than 90% of patients, with pain relief lasting at least six months [50,51]. The complication rate is 2–10%, and 43% undergoing curative ablation showed local progression at a mean time of 4-months follow-up [52,53,54].

##### Cryoablation (CA)

CA utilizes pressurized argon to rapidly freeze tumor tissue, inducing ice-crystal formation that disrupts cellular integrity. The process follows a freeze–thaw cycle with helium gas, enhancing tumor cell apoptosis. Clinical studies report pain relief in 49–100% of patients and no recurrence in 39–92%. Complication rates are low, ranging from 0% to 8% [55,56,57,58].

##### Microwave Ablation (MWA)

MWA heats tissue to 160–180 °C using microwaves to oscillate water molecules, causing coagulative necrosis. Unlike RFA, MWA is less constrained by anatomy and treats larger areas (up to 6 cm). Patients report a 92% average pain reduction within 12 weeks, with fewer complications [50,59,60,61,62].

##### High-Intensity Focused Ultrasound (HIFU)

HIFU involves high-energy ultrasound waves to thermally ablate tumor areas, causing coagulative necrosis. Each treatment targets a small zone, which can be expanded for larger volumes. HIFU is more advanced to superficial bone lesions due to ultrasound absorption by the bone cortex. Studies have reported that HIFU provides effective pain control with minimal complications [53,63,64,65].

##### Electroporation

Electroporation applies high-intensity electric fields to permeabilize cell membranes, inducing apoptosis (irreversible) or enabling drug delivery (reversible). Irreversible electroporation (IRE) creates a precise ablation zone and is advantageous near neurovascular structures due to its non-thermal mechanism. However, clinical data on bone tumors are limited [64,66,67].

#### 5.2.2. Reconstruction Techniques

##### Kirschner Wire and Cannulated Screw Fixation

Kirschner wires have long been used to enhance the periacetabular region, both in open surgery and percutaneously, forming part of the Harrington procedures. More recently, cannulated screws have been applied in this field to provide better biomechanical support. The irregular structure of the periacetabular region makes precise screw placement critical for effectively reconstructing its biomechanical properties.

**Figure 4:**
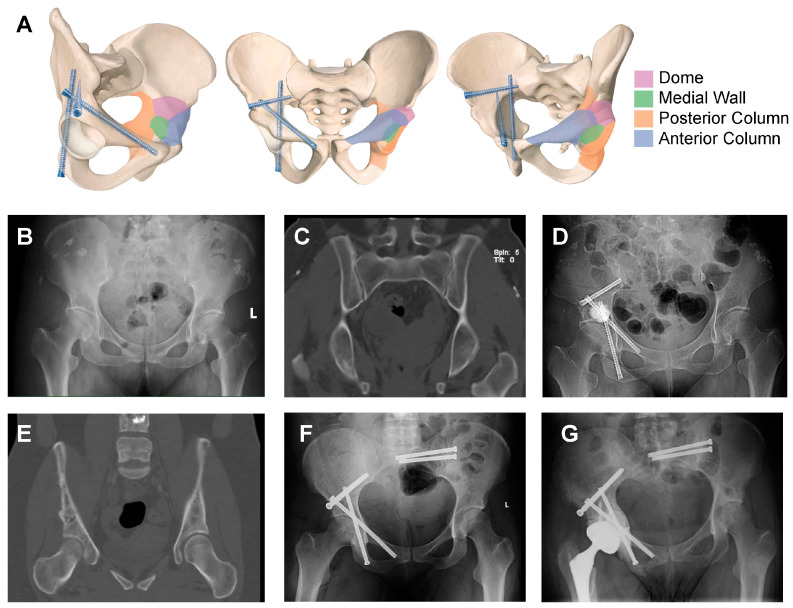
Illustration of TRIPOD techniques. (**A**) Anatomical illustration of the MAC classification and the screw configuration of the TRIPOD technique in different views of the pelvis. A 62-year-old female with metastatic breast cancer. The preoperative pelvic X-ray (**B**) and CT coronal view (**C**) demonstrate a Type 3b lesion in the right pelvis. (**D**) The postoperative X-ray shows treatment with the TRIPOD technique combined with percutaneous bone cement injection into the osteolytic cavity in the periacetabular region. (**E**) A 37-year-old female with multiple metastatic breast cancer; the CT coronal view demonstrates a Type 3b lesion in the right pelvis. (**F**) The postoperative X-ray shows treatment with the TRIPOD technique. (**G**) The X-ray demonstrates the conversion from the TRIPOD technique to a total hip revision performed 14 months after TRIPOD surgery due to tumor progression.

A notable advancement in this field is the Tripod technique, which has demonstrated promising clinical outcomes for acetabular stabilization (Figure 4). This technique conceptualizes the acetabulum as a three-braced tent, where the inferior wall acts as the tent’s apex, while the anterior column, posterior column, and dome form the three structural walls. The technique involves the strategic placement of three screws—anterior column, posterior column, and trans-columnar—configured in a cross-linked manner to mimic a tripod structure, which is known for its geometric stability. The femoral head is supported within this tent-like structure, ensuring enhanced biomechanical stability and load-bearing capacity.

Clinical studies indicate that the Tripod technique is a straightforward and efficient procedure, typically requiring an average operative time of 137 min, with no intraoperative blood loss or significant complications [68]. Patients experience immediate pain relief and improved ECOG performance scores. Additionally, the integration of artificial intelligence (AI)-based preoperative planning and 3D-printing-assisted surgical planning has significantly enhanced the accuracy and efficiency of this procedure. AI-based Deep Neural Network (DNN) models can analyze pelvic CT scans to identify optimal screw entry points and trajectories, ensuring precise placement. The use of personalized 3D-printed surgical guide frames further enhances surgical accuracy, reducing the operative time to under one hour and improving procedural outcomes. These advancements highlight the role of AI and additive manufacturing in improving surgical precision and reducing intraoperative variability [68,69].

##### PMMA Bone Cement

Polymethyl methacrylate (PMMA) bone cement is widely used in orthopedic oncology due to its dual functionality in tumor elimination and mechanical reinforcement. Its application in periacetabular metastatic lesions offers both immediate structural stabilization and local tumor control through thermal necrosis. By inducing coagulative necrosis, PMMA aids in reducing the tumor burden while simultaneously reinforcing weakened bone, making it particularly useful for large osteolytic lesions.

Although PMMA has traditionally been utilized in open surgery, recent advancements have enabled its application in percutaneous procedures, allowing for a minimally invasive approach to acetabular reinforcement. In most cases, PMMA is combined with other techniques, such as screw fixation, to optimize biomechanical stability and prevent progressive collapse. Despite its advantages, PMMA is associated with potential complications, including cement leakage and intra-articular displacement, which necessitate careful procedural planning. Nevertheless, studies have demonstrated its efficacy in providing significant pain relief, improving ambulation, and enhancing the overall quality of life for patients with metastatic bone disease [70,71,72,73,74].

### 5.3. Combination of Minimal Intervention Technique

Minimally invasive techniques, whether applied individually or in combination, have gained significant attention for the management of metastatic periacetabular lesions. Despite encouraging results, there remains no consensus on the optimal treatment strategy. Most available studies are single-institution investigations, the absence of large-scale, multicenter studies and randomized controlled trials limits the standardization of these treatment modalities, making individualized decision-making crucial.

Tumor elimination techniques, such as radiofrequency ablation (RFA) and cryoablation (CA), have been shown to provide substantial symptom relief and improved quality of life [52,53,54]. Similarly, structural reinforcement methods, including Tripod screw fixation and bone cement augmentation, have demonstrated comparable positive outcomes when used independently. The combination of these techniques appears particularly promising. For example, studies highlight the success of integrating tumor ablation with structural reinforcement, such as the internal fixation approach, which offers advantages over open surgery. This combined strategy is simpler, requiring only basic instrumentation and fluoroscopy, and is associated with lower complication rates [75,76,77,78,79].

Interestingly, even without direct tumor elimination, patients treated solely with Tripod fixation have shown significant healing of pathological fractures, with an average follow-up of 13.4 months [68,80]. Many of these patients also received systemic or localized radiotherapy, which likely contributed to the observed bone healing. These outcomes suggest that structural reinforcement alone may suffice in select cases, raising important questions about optimizing treatment strategies. Specifically, the findings emphasize the need to balance avoiding overtreatment with ensuring adequate care, particularly in patients with limited physical fitness or complex metastatic disease. The growing body of evidence highlights the potential of minimally invasive techniques to address diverse clinical scenarios. Further research, including multicenter comparative studies, is necessary to refine patient selection criteria, establish standardized treatment protocols, and determine the optimal combination of minimally invasive and reconstructive interventions. By addressing these gaps, the field can move toward more evidence-based and patient-centered approaches for improving outcomes in periacetabular metastatic disease.

## 6. Conclusions and Future Directions

The primary objectives in managing periacetabular metastatic cancer are to alleviate pain, enhance mechanical stability, repair impaired pelvic force conduction caused by osteolytic lesions, restore mobility and functional status, and facilitate the rapid resumption of systemic cancer treatments. For decades, open surgery has been the standard treatment for pelvic tumor invasion. Techniques such as focal curettage, followed by reconstruction using enhanced cups, total hip prostheses, or modular semi-pelvic prostheses combined with bone cement and screws, have been employed to restore structural integrity. These procedures have demonstrated effectiveness in providing pain relief, enabling weight-bearing and mobility, and achieving relative local tumor control.

However, the invasiveness of open surgery presents significant challenges, particularly for metastatic cancer patients who are typically older, carry a high tumor burden, and may be immunosuppressed due to ongoing systemic treatments. The risks associated with general anesthesia, coupled with substantial intraoperative and perioperative complications, further complicate the decision-making process. Despite observed benefits in pain relief and functional recovery, the high complication rates and prolonged recovery periods often disrupt systemic cancer therapies, necessitating the exploration of alternative approaches.

Minimally invasive surgery (MIS) has emerged as a promising alternative. These techniques integrate tumor ablation with structural reinforcement of the periacetabular region, offering a safer clinical profile. MIS procedures are associated with fewer intraoperative complications, faster recovery, and significant symptom relief while restoring function in most patients.

Given the challenges of conducting randomized controlled trials to determine the optimal treatment strategy for periacetabular lesions, current evidence highlights comparable outcomes between minimally invasive surgery and open surgery. This raises the critical question of how treatment can be best tailored to meet individual patient needs. The primary goals of intervention should focus on pain relief, reducing reliance on analgesics, restoring hip function, enabling patients to resume daily activities, and ensuring uninterrupted systemic cancer treatments, which may ultimately contribute to improved overall survival.

Effective treatment planning requires a multidisciplinary team (MDT) approach, incorporating expertise from orthopedic surgeons, oncologists, and interventional radiologists. When symptomatic periacetabular lesions are detected, patients should be informed about surgical options as complementary to systemic therapy and radiotherapy. Open surgery could be appropriate for patients with extensive osteolytic lesions encompassing the acetabulum or hemipelvis, large painful soft tissue masses, or when simple internal fixation cannot provide sufficient structural support. Open surgery is also indicated as a salvage procedure in cases of complications following MIS or secondary conversion to total hip replacement for patients initially treated with Tripod fixation. Minimally Invasive Surgery, on the other hand, is suitable for a broader spectrum of patients, particularly those with comorbidities, limited life expectancy, or favorable responses to systemic treatment. The selection of a specific MIS technique depends on factors such as tumor size, location, and the extent of osteolytic destruction. While questions remain regarding whether combining multiple techniques provides superior outcomes compared to single modality interventions, promising results from Tripod fixation—demonstrating a high rate of pathological bone healing in conjunction with systemic treatments—highlight its potential as a standalone effective option.

Our review had several limitations. Firstly, there were potential publication biases, particularly in the studies on minimally invasive techniques, where positive outcomes were more likely to be reported, possibly skewing perceptions of treatment effectiveness. Second, the generalizability of current findings is limited, as most studies originate from a few specialized centers, making it difficult to translate results across diverse healthcare settings and patient populations. Lastly, and most importantly, there is a lack of high-quality RCTs and large-scale studies focusing on the surgical management of periacetabular metastases, particularly in terms of perioperative outcomes and long-term prognosis. Consequently, the studies included in this review are primarily retrospective and single-center, restricting the level of evidence to Level IV.

Future directions advancing the management of metastatic periacetabular lesions require strategies that balance tumor control and quality of life. Several key research areas should be prioritized, including oncologic outcomes post-surgery, as different malignancies exhibit distinct patterns of bone metastasis and treatment responses, necessitating stratification by cancer type to refine patient selection criteria and optimize treatment strategies. The integration of minimally invasive techniques should also be further explored, with clinical trials evaluating the efficacy of combining multiple MIS techniques, such as tumor ablation with structural reinforcement, to establish optimal treatment protocols while reducing complications. Additionally, a deeper understanding of the biomechanical properties of periacetabular lesions based on anatomical location is essential for determining the best fixation or reconstruction methodologies. Artificial intelligence (AI) and machine learning algorithms hold significant promise in revolutionizing orthopedic oncology. AI-based systems can analyze real-world clinical data, predict patient-specific treatment outcomes, and optimize surgical planning. The integration of AI-assisted preoperative planning, automated screw trajectory identification, and 3D-printed patient-specific surgical guides has the potential to enhance surgical precision, mitigate human bias, and streamline workflows, ultimately improving clinical outcomes.

By addressing these critical research priorities, the field can move toward evidence-based, personalized approaches for managing periacetabular metastatic cancer, ensuring a balance between preventing overtreatment and delivering adequate care, thereby optimizing patient outcomes and overall quality of life.

## Figures and Tables

**Table 1:** Characteristics of tumor ablation techniques based on tumor size and location.

Tumor Size		Ablation
Tumor < 3 cm	Single, recurring, multiple (<5), tumors	RFA/MWA
	Tumor adjacent to neurovascular bundle	Bipolar RFA/Electroporation
3 cm < Tumor < 5 cm		Bipolar RFA/Electroporation/MWA
Tumor > 5 cm		Multipolar RFA/Electroporation/CA
